# A versatile imaging platform with fluorescence and CT imaging capabilities that detects myeloperoxidase activity and inflammation at different scales

**DOI:** 10.7150/thno.36264

**Published:** 2019-10-12

**Authors:** Cuihua Wang, Benjamin Pulli, Negin Jalali Motlagh, Anning Li, Gregory R. Wojtkiewicz, Stephan P. Schmidt, Yue Wu, Matthias W.G. Zeller, John W. Chen

**Affiliations:** 1 Center for Systems Biology, Massachusetts General Hospital and Harvard Medical School, Boston, MA; 2 Institute for Innovation in Imaging, Department of Radiology, Massachusetts General Hospital, Boston, MA

**Keywords:** myeloperoxidase, imaging platform, enzymatic activity, neutrophil extracellular traps.

## Abstract

Aberrant innate immune response drives the pathophysiology of many diseases. Myeloperoxidase (MPO) is a highly oxidative enzyme secreted by activated myeloid pro-inflammatory immune cells such as neutrophils and macrophages, and is a key mediator of the damaging innate immune response. Current technologies for detecting MPO activity in living organisms are sparse and suffer from any combination of low specificity, low tissue penetration, or low spatial resolution. We describe a versatile imaging platform to detect MPO activity using an activatable construct conjugated to a biotin moiety (MPO-activatable biotinylated sensor, MABS) that allows monitoring the innate immune response and its modulation at different scales and settings.

***Methods:***We designed and synthesized MABS that contains MPO-specific and biotin moieties, and validated its specificity and sensitivity combining with streptavidin-labeled fluorescent agent and gold nanoparticles imaging *in vitro* and *in vivo* in multiple mouse models of inflammation and infection, including Matrigel implant, dermatitis, cellulitis, cerebritis and complete Fraud's adjuvant (CFA)-induced inflammation.

***Results:*** MABS MPO imaging non-invasively detected varying MPO concentrations, MPO inhibition, and MPO deficiency *in vivo* with high sensitivity and specificity. MABS can be used to obtain not only a fluorescence imaging agent, but also a CT imaging agent, conferring molecular activity information to a structural imaging modality. Importantly, using this method on tissue-sections, we found that MPO enzymatic activity does not always co-localize with MPO protein detected with conventional techniques (e.g., immunohistochemistry), underscoring the importance of monitoring enzymatic activity.

***Conclusion:***By choosing from different available secondary probes, MABS can be used to create systems suitable to investigate and image MPO activity at different scales and settings.

## Introduction

Myeloperoxidase (MPO) is an important pro-inflammatory and oxidative enzyme that is secreted by neutrophils and inflammatory monocytes/macrophages to generate reactive oxygen species (ROS) 1-4. While this is crucial for defense against pathogens 5-7, misdirected increased MPO activity has been linked to detrimental effects on the host in a large number of diseases including atherosclerosis 8, 9, Alzheimer' disease 10, 11, stroke 12, multiple sclerosis 13, 14, non-alcoholic steatohepatitis 15, and atrial fibrillation 16, among others. MPO has also been implicated in neutrophil extracellular trap (NET) formation 17, 18, where bacteria are entrapped in chromatin-granule protein webs and subsequently killed 19. In addition to its functions as an oxidative enzyme, pro-inflammatory non-enzymatic effects have also been reported 20, 21. While many molecules involved in the inflammatory cascade have both beneficial and damaging effects depending on the microenvironment, MPO is only expressed by pro-inflammatory cells and the oxidative radicals it generates are unequivocally damaging to biological tissues 1, 22.

In the absence of inflammatory stimuli, MPO is stored in myeloid cell granules, but is readily secreted into the extracellular space upon appropriate stimulation 23. Thus, measuring MPO protein, which is often performed via antibody-mediated extraction from tissue homogenates 24 or immunohistochemical techniques that involve tissue permeabilization, may not result in representative results since it cannot distinguish inactive stored intragranular MPO from secreted active MPO. Measuring MPO activity in living tissue circumvents these issues, but imaging MPO activity with high spatial resolution and high specificity is challenging. Several MPO imaging agents have been developed, such as luminol 25, 26, bioluminescent L-012 27, oxazine conjugated nanoparticles 28, SNAPF (which detects HOCl but not MPO activity directly) 29, and 5HT-Cy3 30. However, low signal penetration and/or lack of specificity have so far resulted in limited use of these agents for MPO activity. The magnetic resonance imaging (MRI) probe MPO-Gd 31, 32 has been validated in animal models of inflammation (e.g. 33-36), but MR imaging lacks spatial resolution at the cellular level. Furthermore, MRI is relatively expensive and not widely available for animal research purposes.

In this study, we report the development of an MPO activatable biotinylated sensor (MABS) platform and demonstrate that this platform exhibits high utility for imaging applications in living animals as well as in tissue sections. By employing a pre-targeting approach, in which MABS functions as a targeting agent that can be activated directly by MPO and is retained at the site of inflammation through protein binding, subsequent administration of reporter-tagged streptavidin then binds to the biotin moiety of MABS to provide visualization. We illustrate the flexibility of the platform by generating fluorescence and CT imaging agents that detected MPO activity at microscopic and macroscopic scales to monitor the changes in the innate immune response. We validated the specificity of the MABS platform for MPO *in vitro* against other peroxidases, and *in vivo* using MPO inhibition and MPO-deficient mice in multiple models. This platform enabled differentiation of active from inactive MPO, revealing primed vs. pathological innate immune response that had not been previously available.

## Methods

### Synthesis of MPO Activatable Biotinylated Sensor (MABS) and Nonspecific Analog (Fig. 1b)

All chemicals were obtained from Sigma Aldrich unless otherwise stated. 5-hydroxytryptamine was obtained from Alfa Aesar (Ward Hill, MA).

*Synthesis of compound** 2**.* To the solution of K_2_CO_3_ (440 mg, 3.2 mmol) in water (4mL) L-5-hydroxy-tryptophan (5-HTP, 330 mg, 1.5 mmol) was added. Then, a solution of di-t-butyl dicarbonate (392 mg, 1.8 mmol) in THF (2 mL) was added to the above solution. The reaction was stirred at room temperature for 2 h. The reaction was neutralized to pH 2-3 by 1M HCl. After evaporating to remove THF, the solution was extracted by ethyl acetate (10 mL x 3), the organic phase washed by brine (5 mL x 3), dried over anhydrous Na_2_CO_3_, and evaporated. The residue underwent flash column (EA as eluent) to give compound **2** (76%).

*Synthesis of compound*** 3**. To the solution of compound **2** (192 mg, 0.6 mmol) in DMF (3 mL) was added EDC.HCl (140 mg, 0.72 mmol), subsequently HOBt (108 mg, 0.72 mmol), and the mixture was stirred for 10 min. Then the solution of 5-hydroxytryptamine (110 mg, 0.5 mmol) in DMF (2 mL) prepared from its hydrochloride salt by adding triethylamine (140 μL, 1 mmol) was added to the above mixture. The reaction was stirred for another 2 h. The reaction was extracted with ethyl acetate (10 mL x 3), the organic layer washed by brine (5 mL x 3), dried over anhydrous Na_2_CO_3_, and evaporated. The residue underwent flash chromatography (EA as eluent) to give the white solid (196 mg) in the yield of 82%.

*Synthesis of compound****1*** *(MABS).* To the solution of 10% TFA in DCM (2 mL) was added compound **3** (95 mg, 0.2 mmol), and the reaction was stirred for 5 h at room temperature. Then the reaction was evaporated to remove the solvent and put to the next step without further purification. To the solution of the obtained compound in DMF (2 mL) was added TEA (140 uL, 1 mmol) then biotin-NHS (54 mg, 0.16 mmol). The reaction was stirred for 4 h at room temperature. The reaction was purified through preparative HPLC (gradient: 0-100% of acetonitrile/water) to give the final compound (36 mg, 40%).

The non-specific analogue was synthetized using identical steps except for that L-tryptophan and tryptamine were used instead of 5-HTP. The indole ring of the tryptophan/tryptamine cannot be oxidized by MPO.

Each final compound was characterized by ^1^H, ^13^C NMR and LC-MS (see Supplementary Material).

### *In Vitro* MABS oligomerization and Protein Binding

MABS (1 mM) in PBS (100 μL of total volume) was incubated with MPO (6 μL, 1,080 units/mg protein, Lee Biosolutions, Maryland Heights, MO), glucose oxidase (GOX, 4 μL, 1 mg/mL, 100 units/mg protein, Sigma-Aldrich, MA) and glucose (1 M, 4 μL, Sigma-Aldrich, MA) at 37 ^o^C for 15 min. 100 μL of methanol was added to the suspension, which was centrifuged and subjected to high resolution liquid chromatography (LC-MS, Thermo Q-Exactive Plus mass spectrometer, MA).

90 μL Matrigel (BD Bioscience, San Jose, CA) and 30 μL minimum essential medium (MEM, Corning) with or without purified MPO (5 μL) and glucose oxidase (4 μL, 1 mg/mL) at 4 ^o^C were added to each well of a 96-well plate and incubated for 40 min at 37 ^o^C. 50 μM of the fluorescent probe in 120 μL MEM was loaded on top of the gel and incubated for 1h. After removing the upper solution, the remaining gel was washed three times with PBS. Different amounts of SAF-647 in 120 μL MEM were added to the gels and incubated for another 1h. The upper solution was removed and the gel was washed three times with PBS. The fluorescent signal was then detected with a fluorescence reflectance imaging (FRI) system (OV-110, Olympus, Tokyo, Japan) at 595-635 nm excitation and 675/50 nm emission.

For Fibrinogen binding experiments, Fibrinogen (5 mg/mL, Sigma-Aldrich) in PBS (40 μL) with glucose oxidase (GOX, 1 mg/mL, 2 μL, Affymetrix, Santa Clara, CA), glucose (4 μL, 1 M) and MABS (40 μM) was incubated with MPO (4 μg, 2 mg/ml, Lee Biosolutions, Maryland Heights, MO), EPO (2.6 μg, Lee Biosolutions, MO) or LPO (5.7 μg, Sigma-Aldrich) for 1 h at 37 ^o^C, and experiment with no enzyme added as control. Equivalent activities of MPO, LPO, and EPO were determined with the guaiacol activity assay 37. The reaction mixture was filtered through Biospin P-6 columns (Bio-Rad, CA) to remove unactivated MABS. Then streptavidin (1 μL, 2 mg/mL) was added to the above filtrate (20 μL) and incubated for 30 min at 37 ^o^C. 4 μL of the above sample was loaded onto the 7% of native gel followed by native-PAGE. The gel was rinsed with DI water and stained with EZBlue^TM^ gel staining agent (Sigma-Aldrich) for 1 h. The gel was scanned with an HP Scanjet G4050.

### Animals and Study Approval

Four to ten weeks old female C57BL/6J or MPO-knockout mice (from Jackson Laboratories, Bar Harbor, ME) were used for all animal experiments. This study was approved by and in compliance with our Institutional Animal Care and Use Committee.

### Biodistribution, Half-life, and Plasma Stability

To evaluate organ distribution, C57BL/6J mice were injected 2 μL of MABS (10 mM stock solution in DMSO) in 100 μL PBS and after one hour 25 μL of SAF-647 (2 mg/mL, SAF-647, Invitrogen, Carlsbad, CA) was injected intravenously. 30 minutes later the major organs were collected. Biodistribution was measured with a dedicated FRI system, similar to described elsewhere 38. Organs were harvested, weighed, and imaged with FRI. Fluorescence signal was normalized to organ weights and reported as RFUs/mg. Infected brain tissue (see Salmonella cerebritis below) was used as reference. To assess half-life, 2 mM of MABS (150 μL PBS, 5% DMSO) was injected intravenously by tail vein injection into female C57BL/6J mice (n=6) and the blood was taken at 0 min (pre-injection), 1 min, 3 min, 5 min, 10 min, 15 min, 30 min, 1 h, 3 h, and 8 h, respectively. The blood collected was centrifuged at 2,000 G for 15 min at 4 ^o^C to obtain the plasma. 60 μL methanol was added to each plasma sample (20 μL). After 5min, the plasma was centrifuged (1,500G, 10min), and the UV absorption of each sample was measured with UV-visible spectrophotometer (Varian Cary Bio 50, Agilent Technologies, Santa Clara, CA) at a wavelength of 275 nm. To assess plasma stability of MABS, mouse plasma was incubated with MABS (1 mM) and an inference compound (non-specific analogue, 1 mM) at 37 ^o^C for 0, 30 min, 1 h, 2 h and 4 h, respectively. The plasma samples (5 μL) were treated with acetonitrile (30 μL) for 5 min, centrifuged, and the supernatants were subjected to liquid chromatography mass spectrometry (LCMS) analysis (Waters, MA, USA). The areas under curve (AUC) of MABS and the inference peaks at 254 nm were integrated and the ratio of AUC_MABS_ and AUC_inference_ at different time points were compared.

### *In Vivo* Validation of Sensitivity and Specificity of MABS

C57BL/6J and MPO-knockout (KO) mice were fed with normal chow or bioin-free diet for 5 days before imaging with fluorescence molecular tomography (FMT) or fluorescence reflectance imaging (FRI). To validate sensitivity of MABS, we embedded different concentrations of purified human MPO (Lee Biosolutions) and glucose oxidase (GOX, as a H_2_O_2_ donor) in a 1:1 mixture of matrigel (BD Bioscience) and MEM (Corning), and injected 300 μL of this mixture into the subdermal aspect of the thigh. After 30 min, 2 μL of MABS as above was injected intravenously. One hour later 25 μL of SAF-647 (2 mg/mL, SAF-647, Invitrogen, Carlsbad, CA) was injected intravenously, and FMT was performed every 15 minutes for a total of 60 minutes. Mice were imaged on a dedicated FMT system (Perkin Elmer, Waltham, MA) at 635 nm excitation and 655 nm emission.

To validate specificity of MABS, we injected mice with a 1:1 mixture of MEM and Matrigel as above, containing a combination of GOX, MPO, and/or 4-aminobenzoic acid hydrazide (ABAH, a specific irreversible MPO inhibitor 39, Sigma). In addition, ABAH was injected intraperitoneally (i.p.) in some mice. MABS or nonspecific analogue (2 μL of 10 mM stock solution in 100 μL PBS were injected intravenously 30 minutes later, and 25 μL SAF-647 (2 mg/mL) was injected 60 minutes thereafter. Imaging using a dedicated FRI system (Olympus OV-110, Tokyo, Japan) was performed 30 minutes after injection of SAF-647. SAF-647 bound to MPO-sensor was detected at 595-635 nm excitation and 675/50 nm emission.

### Paw Inflammation Fluorescence Imaging

Female C57BL/6J and MPO-KO (both from Jackson laboratories) mice were treated topically with 0.08 μmol phorbol 12-myristate 13-acetate (PMA, Sigma, St. Louis, MO) on one hind paw and with vehicle on the other to induce irritant contact dermatitis. This model is well described in the literature and triggers rapid inflammation with influx of neutrophils into the skin 40-42. 6 h after induction, mice were injected intravenously with either 2 μL of MABS or non-specific analogue as above. 1 h later, 25 μL SAF-647 were injected intravenously to bind to the biotinylated MABS. After 30 min, mice underwent *in vivo* imaging using a dedicated *in vivo* FRI system as above.

### Paw Inflammation CT imaging

Female C57BL/6J and MPO-KO mice (both from Jackson laboratories) were injected subcutaneously a 1:1 emulsion of CFA (1mg/ml, Sigma, St Lous, MO) and saline (40 μL of total volume) on the ventral side of the rear paw under anesthesia with isofluorane. Saline (40 μL) was injected to the opposite side as control. After 24 h, 2 μL of MABS in100 μL PBS was injected via tail vein followed by administration of 200 μL of streptavidin-conjugated gold nanoparticles (GNPs, 0.05% (w/v) gold, Nanocs Inc., NY) after 30 min. The mice were scanned before injection of GNPs (pre-contrast) and at 15, 30 and 60 min post-injection using a Siemens Inveon CT scanner. The CT X-ray source used an 80 kVp and 500 μA tube set at 60 kVp and 500 μA (The k-edge of gold is 80.7 kVp which is beyond the capability of the CT X-ray source of 80 kVp. In order to increase the probability of photoelectric absorption of X-rays from the gold nanoparticles and increase the contrast capability of our agent, we lower the X-ray energies by lowering the tube voltage to a point where a majority of X-rays generated are in the range 25-51 kV range 43-45) with an exposure time of 425 ms per projection over 360 projections and reconstructed by a modified FeldKamp Conebeam reconstruction algorithm (COBRA, Exxim Inc.) into isotropic 90 μm voxels. The images were analyzed using the Horos DICOM viewer (https://www.horosproject.org). Regions of interest (ROIs) were drawn manually around the swollen areas of the hind paws.

### Salmonella Cerebritis Imaging and Immunohistochemistry

Female C57BL/6J and MPO-knockout mice were anesthetized and fixed in a stereotactic head frame (David Kopf Instruments, Tujunga, CA). 1- 3 × 10^6^ colony forming units (CFU) salmonella bacteria (ATCC #14028) were suspended in 2 µL PBS and slowly injected into the deep frontal white matter (2.0 mm lateral and 1.2 mm anterior to bregma) at a depth of 3.0 mm. 23 h later, mice were injected intravenously with 2 µL of MABS, followed by intravenous injection of SAF-647 as above described. Brains were harvested and cut into 2 mm coronal slices using a brain slicer (Harvard Apparatus, Holliston, MA) and immediately underwent FRI as described above. The brain slices were fixed in 4% PFA for 1 h, embedded in OCT (Sakura, Torrance, CA) and snap-frozen in chilled isopentane (Sigma). Serial 7 µm sections were cut on a cryostat (Thermo scientific). Sections were thawed at room temperature for 10-20 minutes, then rehydrated in PBS for 10 min. After they were blocked in blocking buffer (1% of horse serum, 1% bovine serum albumin, 1% normal donkey serum, and 0.3% Triton X-100 in PBS) for 30 minutes at room temperature, the sections were incubated with anti-MPO antibody (1:500: rabbit polyclonal, catalog # RB373, Thermo Scientific, Waltham, MA) diluted in incubation buffer (1% bovine serum albumin, 1% normal donkey serum, and 0.3% Triton X-100) overnight at 4 °C, then incubated with Texas Red -labeled anti rabbit IgG 1:800 for 1 h in room temperature. The above sections were counterstained with DAPI (4', 6-diamidino-2-phenylindole, Invitrogen) and mounted with anti-fade mounting medium. The images were acquired with a 25x objective using Zeiss LSM800-Airyscan confocal microscope. The number of the pixels positive for MABS and MPO protein were counted using ImageJ (version 1.52). Three separate sections were evaluated per animal, and the results were averaged for each animal prior to statistical comparison.

### Bacterial cellulitis and NET imaging

Female C57BL/6J mice and MPO-knockout mice were injected subcutaneously at the dorsal aspect of the thigh with 10^8^ CFU of streptococcus pneumonia (SPn, ATCC #6303) to induce bacterial cellulitis. 6 h after induction, mice were injected intravenously with 2 μL of MABS as above. 1 h later, 25 μL SAF-647 were injected intravenously to bind to MABS. To distinguish neutrophil granule release (where MPO and other granule proteins are secreted into the phagosome or extracellular space) from NET formation (where chromatin strands with MPO are released by neutrophils), we injected 1 μg of the membrane-impermeable DNA dye Sytox Green (Invitrogen) intravenously at the same time. Mice then underwent *in vivo* imaging using a dedicated FRI system as above. SAF-647 bound to MPO-sensor was detected at 595-635 nm excitation and 675/50 nm emission. Sytox Green was detected at 460-490 nm excitation and 530/40 nm emission.

### Statistical Analysis

Statistical analysis was performed using Prism 5.0 software (Graphpad, La Jolla, CA) P-values <0.05 were considered significant. Data were compared using the Student's t test or Mann-Whitney U test. Correlation was determined by calculating the Pearson's correlation coefficient. Fluorescence intensity was quantified using ImageJ software, and results presented as relative fluorescence units (RFU). Data of blood half-life was processed with Prism 5.0 software using two-phase exponential decay model.

## Results

### Design, synthesis and characterization

**Figure 1:**
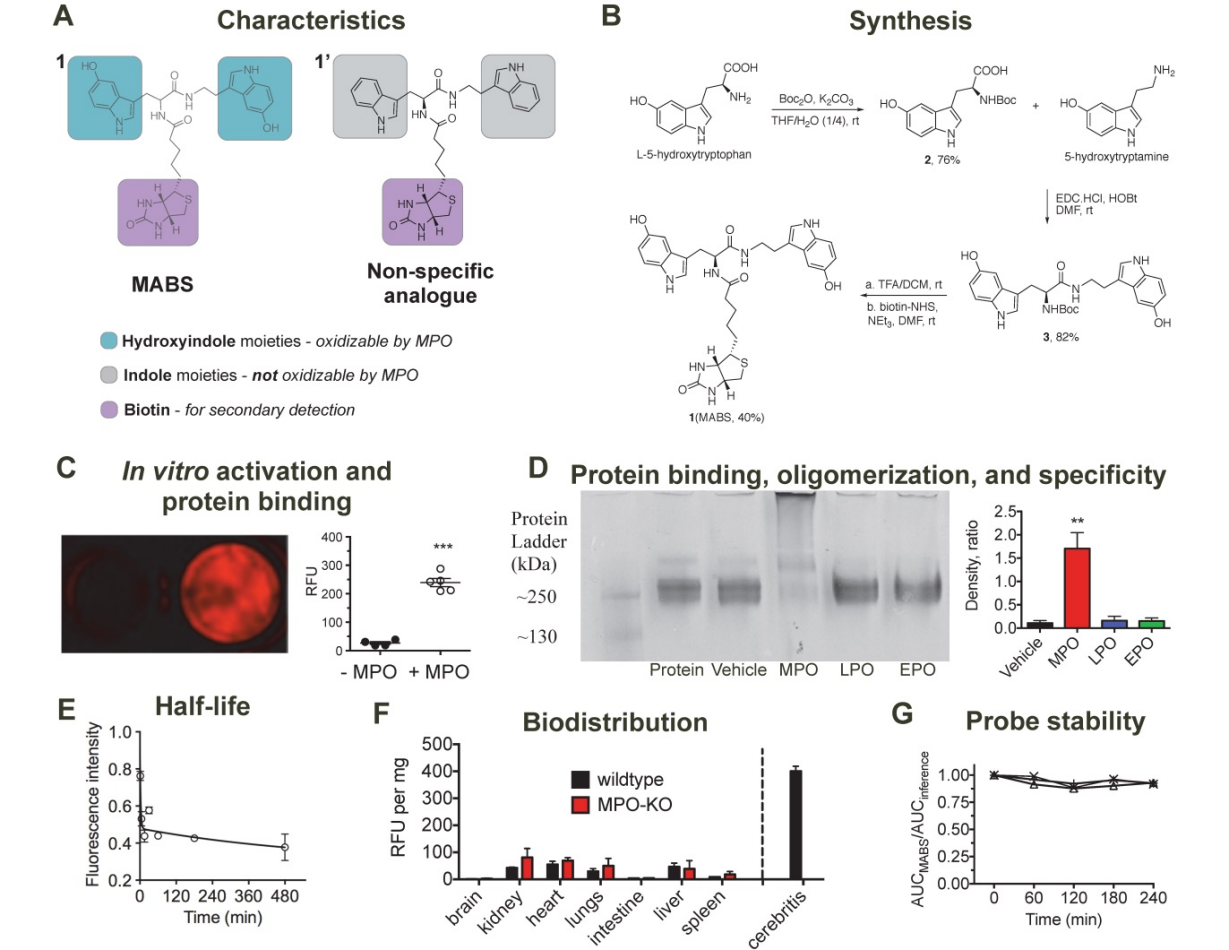
** Synthesis, characterization, *in vitro* validation, and blood half-life of MPO activatable biotinylated sensor (MABS). (A)** Characteristics of the MABS and nonspecific analog. MABS consists of two functional parts: 1) dual 5-hydroxyindole moieties (green) that can be oxidized in the presence of active MPO, which results in protein binding and thus probe retention at the site of MPO activity and 2) a biotin moiety (magenta) that is used for detection with a secondary probe. The nonspecific analog contains indole moieties (gray) instead of 5-hydroxyindole moieties, which cannot be oxidized by MPO. **(B)** Synthesis of MABS. **(C)** Matrigel containing MABS, glucose oxidase, and streptavidin AlexaFluor-647 (SAF-647) without (left well) and with MPO (right well) (*** p < 0.001 by Student t-test. Experiment was replicated twice). **(D)** Native PAGE of fibrinogen incubated with MABS, glucose oxidase and either MPO, LPO, EPO, or vehicle. A high molecular weight band is only seen in the presence of MPO, which suggests fibrinogen multimer formation after binding of MPO-oxidized MABS. **(E)** The blood half-life of the probe is 1.6 min (R^2^ = 0.74). N = 6, experiment was performed once.** (F)** *Ex vivo* organ fluorescence in wildtype and MPO knockout mice injected with MABS and SAF-647 and sacrificed at 30 min to measure biodistribution by fluorescence intensity normalized to organ weights. Infected brain tissue (cerebritis) is shown for reference. **(G)** Plasma stability (N = 3).

MABS consists of two functional parts (**Fig. 1A**): (1) dual 5-hydroxyindole (5-hydroxytryptophan (5-HTP) and 5-hydroxytryptamine (5-HT)) moieties that can be oxidized in the presence of active MPO, which results in protein binding and thus probe retention at the site of MPO activity, (2) the biotin moiety, which can be targeted with a secondary detection probe. A nonspecific analog that contains indole (tryptophan/tryptamine) moieties instead of the 5-hydroxyindole moieties was also synthesized as negative control, since the indole ring of the nonspecific analogue cannot be oxidized by MPO and thus the probe is not retained at sites of MPO activity.

Synthesis of MABS is shown in **Fig. 1B** and described in detail in the methods section. In brief, starting with 5-HTP, Boc-protection resulted in compound **2**, which was then coupled with 2-hydroxyindole acetic acid using 1-ethyl-3-(3-dimethylaminopropyl)-carbodiimide hydrochloride (EDC.HCl) and N-hydroxysuccinimide (NHS) as coupling agents, resulting in compound **3**. De-protection of the Boc group under acidic conditions and coupling with biotin-NHS resulted in the final MABS with an overall yield of 25%. Each final compound was characterized by ^1^H, ^13^C NMR and LC-MS (see Supplementary Material).

### Probe oxidation by MPO results in oligomerization and protein binding and shows specificity over other peroxidases

We hypothesized that MABS would form oligomers and/or bind to proteins after oxidation by MPO. First, we incubated MABS with MPO in the presence of glucose oxidase as an H_2_O_2_ donor and detected the oligomers formed by high-resolution liquid chromatography mass spectrometry (LC-MS). We identified dimers and trimers derived from MABS after activation (**Suppl. Fig. S1**). Similarly, we incubated Matrigel, MABS and GOX with or without MPO. Following washing steps to remove unbound probe, and incubation with streptavidin AlexaFluor-647 (SAF-647), we detected increased fluorescent signal in the presence of MPO but not without it (**Fig. 1C**). In a subsequent experiment utilizing fibrinogen as the binding protein, MABS was incubated with or without MPO, lactoperoxidase (LPO), or eosinophilic peroxidase (EPO). Only after the addition of MPO but not LPO or EPO did we observed an additional band above fibrinogen (**Fig. 1D**), which is consistent with fibrinogen multimers as a result of activated MABS binding to the protein.

### Biodistribution and plasma stability of MABS

MABS was intravenously administered to C57BL/6 mice followed by SAF-647, and mouse blood samples were collected before and at different time points after injection of MABS. To calculate the blood half-life of MABS, the absorption intensity of plasma at wavelength of 275 nm was measured and found to be 1.6 min with a tissue distribution phase of 425.5 min (R^2^ = 0.74, **Fig. 1E**) calculated with a two-phase decay model. To assess biodistribution, organs were harvested, fluorescent signal quantified with fluorescence reflectance imaging (FRI), (**Fig. 1F**) and signal normalized to organ weight 38. Slightly higher fluorescent signal was found in kidneys, liver, heart, and lungs, while low signal was detected in brain, intestine, and spleen. There was no significant difference between the biodistributions of wildtype and MPO-KO mice. In addition, even organs with higher signal demonstrated approximately 5-fold lower signal than that of infected brain tissue, which is shown as reference. When incubated in mouse plasma, MABS was stable up to 4 hours (**Fig. 1G**).

### Sensitivity and specificity *in vivo*

**Figure 2:**
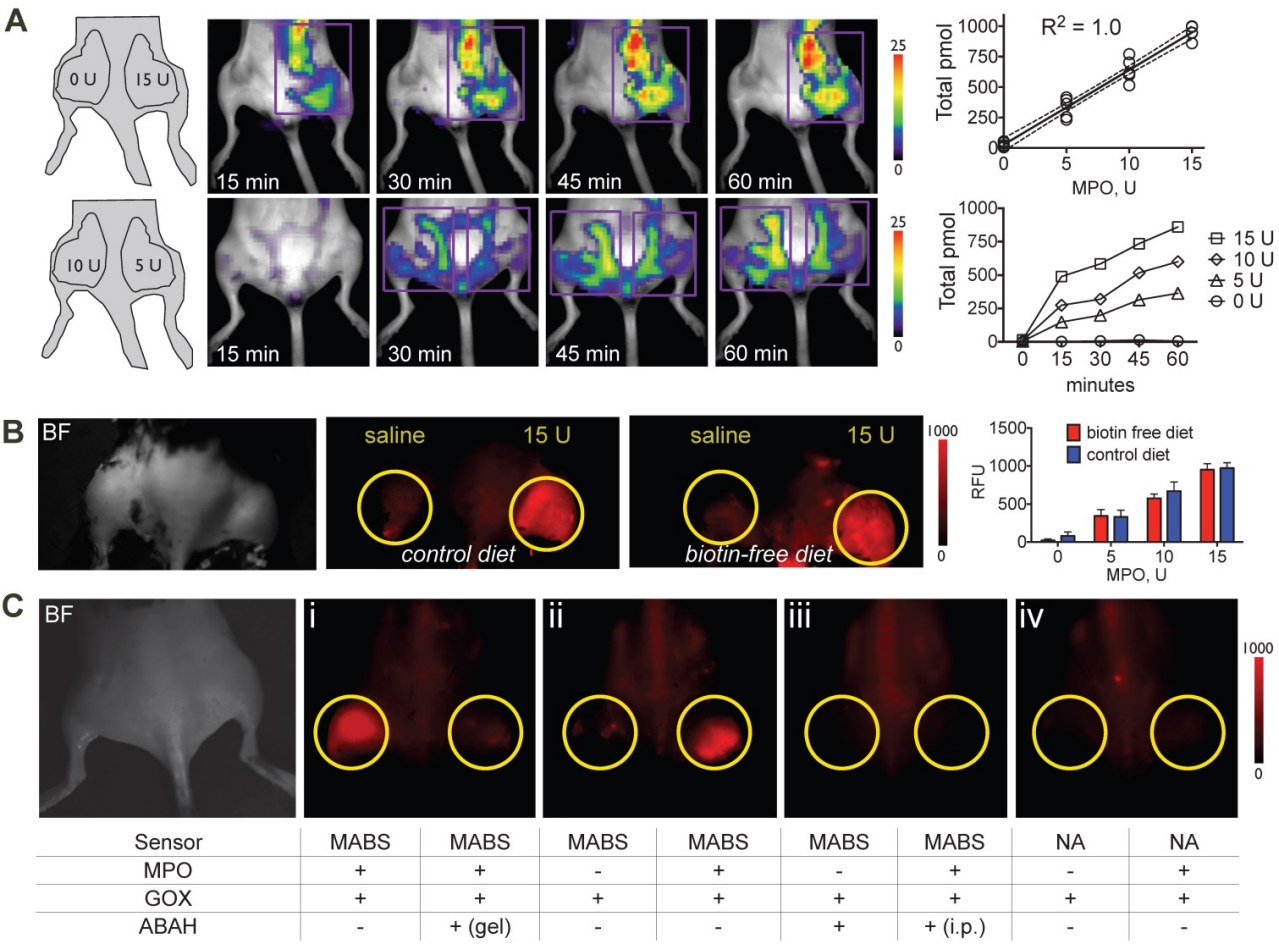
** Sensitivity and specificity of MPO activatable biotinylated sensor (MABS) *in vivo*.** (**A**) Fluorescence molecular tomography (FMT) at 15, 30, 45, and 60 minutes after injection of MABS and streptaividin-AlexaFluor-647 (SAF-647), as well as different concentrations of MPO embedded in Matrigel into mouse thighs. A schematic on injection sites and quantities of MPO is seen in the left panel. Purple rectangle outlines the region of interest (ROI). Image quantification revealed a linear increase (R^2^ = 1.0, p=0.004) with increasing quantities of MPO, with increasing of fluorescence signal over 60 minutes (N = 4, experiment was performed once). (**B**) Fluorescence reflectance imaging (FRI) of mice fed either biotin free diet or normal chow for 5 days, after injection of MABS and SAF-647, as well as different concentrations of MPO embedded in Matrigel into mouse thighs. No significant difference in signal or background is noticed between mice on biotin-free diet and mice on normal chow (N = 3 per group, one-way ANOVA was used to determine statistical significance, experiment was performed once) (**C**) FRI of different combinations of glucose oxidase (GOX), MPO, and the irreversible MPO inhibitor ABAH embedded in Matrigel into mouse thighs and injected with MABS and SAF-647 (panels ii-iv). Yellow circles outline sites Matrigel injection. ABAH was either embedded in Matrigel together with MPO and GOX, or administered intraperitoneally (i.p.). Nonspecific analogue (NA) was also injected into some mice (panel iv). Brightfield image indicating mouse positioning is shown in the left panel (N = 4, experiment was replicated thrice).

To verify sensitivity of the probe *in vivo*, we embedded different concentrations of MPO in Matrigel and injected Matrigel mixtures subcutaneously into the thighs of C57BL/6 mice that had been kept on either normal chow and biotin-free diet for 5 days, followed by injection of MABS and SAF-647 and quantitative fluorescence molecular tomography (FMT) imaging. We detected a linear increase in signal with increasing concentrations of MPO, while no signal over background was detected without MPO (**Fig. 2A**). Furthermore, to evaluate if endogenous biotin in mice would affect the potency of MABS in *in vivo* imaging applications, we performed the MPO implant experiment in mice fed with normal chow that contains biotin and with biotin-free chow to minimize biotin in the mouse. We found no difference between mice on normal chow compared to those on biotin-free diet (**Fig. 2B**), and thus, all subsequent experiments were performed on mice fed normal chow.

To verify specificity of the probe, we embedded different combinations of GOX, MPO, and the specific irreversible MPO inhibitor 4-aminobenzoic acid hydrazide (ABAH) in Matrigel into mouse thighs, followed by injection of MABS or the nonspecific analogue, and SAF-647 as above. Mice then underwent FRI. We detected increased fluorescent signal with MPO and GOX, which was readily inhibited by the addition of ABAH to Matrigel (**Fig. 2C, panel I** and** Fig. S2**). With GOX but without MPO, no fluorescence signal was detectible, proving that MABS is not activated by H_2_O_2_ alone (**Fig. 2C, panel ii** and** Fig. S2**). As expected, systemic (intraperitoneal) injection of ABAH also resulted in the abrogation of fluorescence signal consistent with successful MPO inhibition (**Fig. 2C, panel iii** and** Fig. S2**). Lastly, the nonspecific analog was insensitive to both GOX and MPO (**Fig. 2C, panel iv** and** Fig. S2**).

### Imaging of inflammation

**Figure 3:**
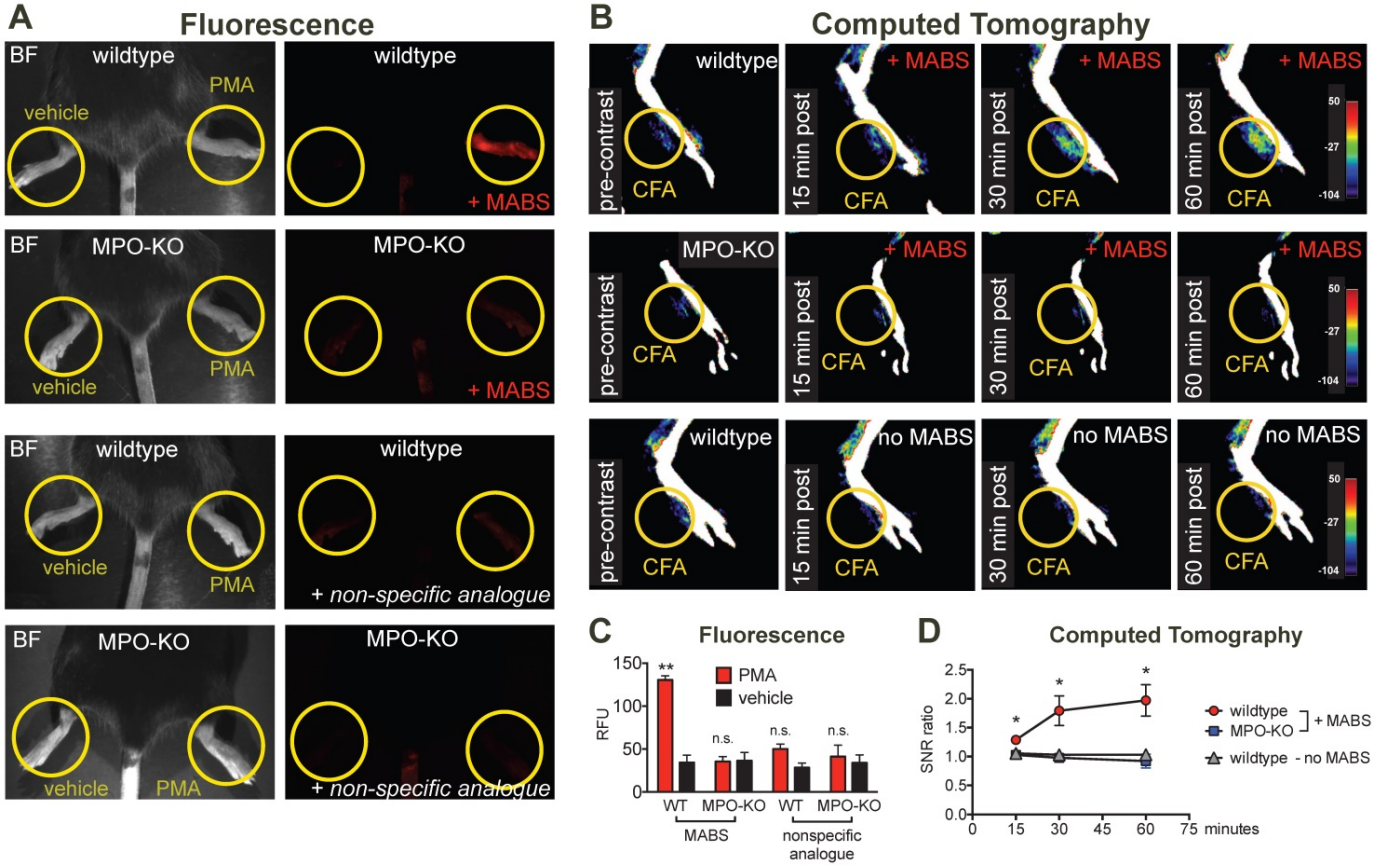
** MPO fluorescence and CT imaging of paw inflammation.** (**A**) Mice were treated with PMA to induce inflammation on the right hindpaw, and vehicle as negative control on the left hindpaw, and fluorescence reflectance imaging (FRI) was performed. Yellow circles outline sites of topical administration of PMA or vehicle. Brightfield images are presented to outline anatomy (left column). Fluorescence images of MPO activity demonstrate increased fluorescence signal in the PMA-treated right hindpaw in a wildtype mouse injected with MPO activatable biotinylated sensor (MABS) (top row). In an MPO-KO mouse injected with MABS (second row), a wildtype mouse injected with non-specific analogue (third row), and an MPO-KO mouse injected with non-specific analogue (bottom row) no fluorescence signal over background was detected in either hindpaw. (**B**) Mice were treated with Complete Freud's Adjuvant (CFA) to induce inflammation on the right hindpaw, and saline as negative control on the left hindpaw. 24 hours later, CT imaging before and at 15, 30 and 60 minutes after intravenous injection of streptavidin-gold conjugated nanoparticles was performed. Yellow circles outline sites of CFA or saline administration. A wildtype mouse injected with CFA in the left hindpaw demonstrates contrast enhancement at the injection site over time (top row). No contrast enhancement is seen in an MPO-KO mouse injected with CFA in the left paw (middle row), or in a wildtype mouse with CFA in the left paw injected with streptavidin-conjugated GNPs only without MABS (bottom row). (**C**) Quantification of fluorescence signal in the hindpaws of PMA and vehicle-treated mice (** p < 0.01, n.s. not significant, N=3 mice per group, student's t-test, experiment was replicated twice). (**D**) Quantification of contrast enhancement in the hindpaws with CFA in wildtype and MPO-KO mice with MABS and wildtype mice with CFA with gold nanoparticles only, respectively. (* p < 0.05, N=3 mice per group, student's t-test experiment was performed once).

For *in vivo* applications in disease models, we first studied specificity of the probe in irritant contact dermatitis. When wildtype mice were treated topically on the hindpaw with PMA to induce irritant contact dermatitis (**Fig. 3A**), increased fluorescence signal on the PMA-treated hindpaw but not the vehicle-treated hindpaw was observed. In MPO-KO mice treated with PMA and injected with MABS, no fluorescence over background could be detected. Similarly, injection of the nonspecific analogue into PMA-treated wildtype mice and MPO-KO mice did not result in fluorescence over background (quantification of fluorescence signal is shown in **Fig. 3C**).

We next studied applicability of MABS for molecular CT imaging. We induced paw inflammation by injecting Complete Freud's Adjuvant (CFA) emulsion into the left ventral hindpaw, and normal saline as control into the right hindpaw. CT images before and at 15, 30, and 60 minutes after intravenous injection of streptavidin-conjugated gold nanoparticles (GNPs) were acquired on a dedicated small animal CT scanner (**Fig. 3B** and** Fig. S3**). Contrast enhancement on the left hindpaw with CFA was detected over the time course of 60 min in wildtype mice, but not those in wildtype mice with GNPs only, or in MPO-KO mice. No contrast enhancement above background was seen on the right hindpaw with saline. Quantification of contrast enhancement can be seen in **Fig. 3D**.

### Imaging of Infection

**Figure 4:**
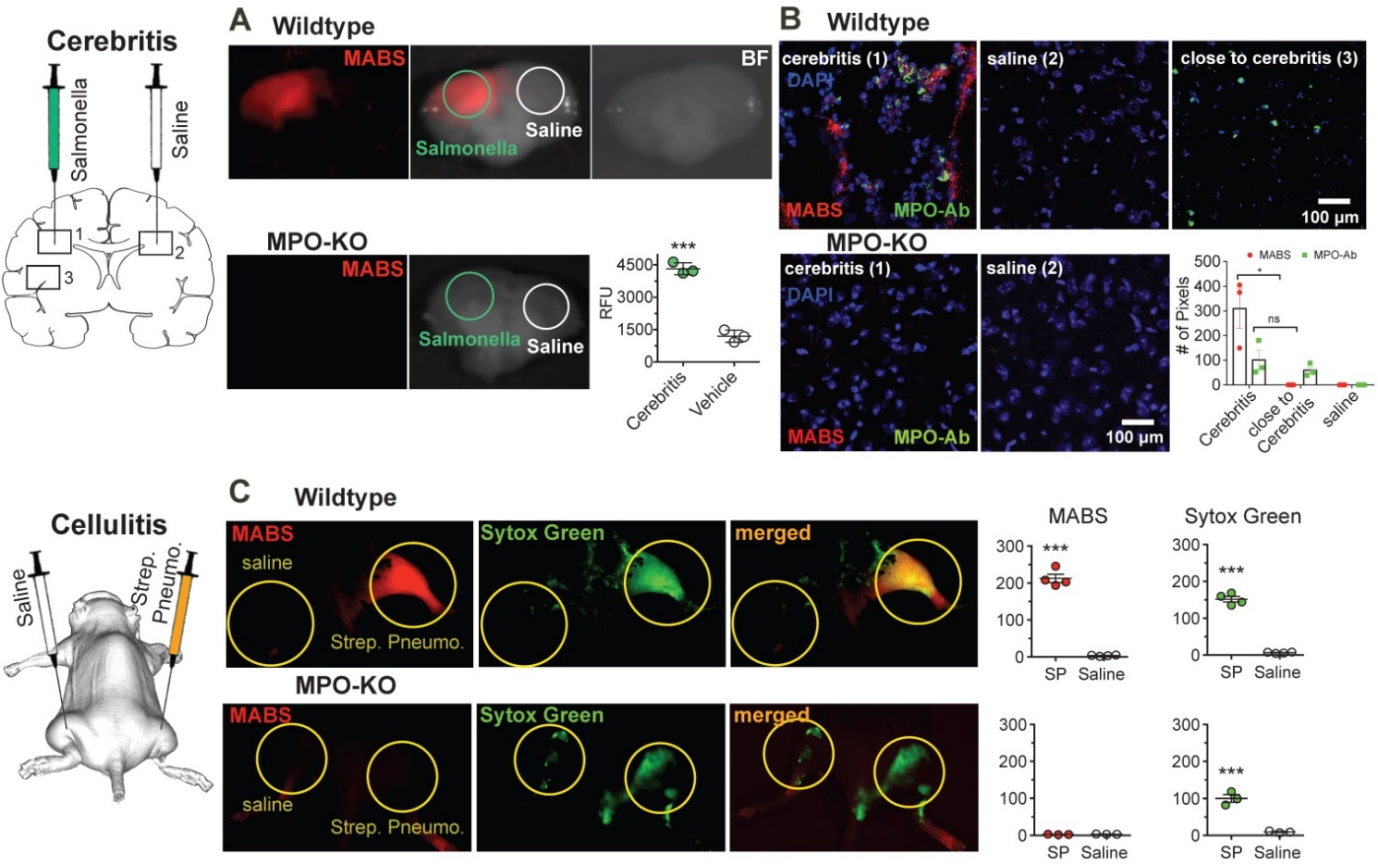
** MPO fluorescence molecular imaging in infection.** (**A**) Mice were injected with salmonella intracerebrally to induce cerebritis. Fluorescence reflectance imaging (FRI) of coronal brain slices was performed, showing increased fluorescence signal consistent with MPO activity in the ipsilateral but not contralateral hemisphere. Saline injection did not trigger significant MPO activity (N = 6, *** p < 0.0002, student's t-test, experiment was performed twice). (**B**) Immunofluorescence microscopy of fresh-frozen tissue sections. Staining for MPO activity (MABS, red) and MPO protein (as detected with an MPO-antibody, green) revealed increased MPO protein both within and close to the regions of cerebritis, while elevated MPO activity was only detected within the region of cerebritis (bar = 100 μm, confocal microscopy at 25X). (**C**) Wildtype and MPO-KO mice were injected subcutaneously with Streptococcus pneumoniae (SPn) to induce bacterial cellulitis with formation of neutrophil extracellular traps (NETs), or with saline as negative control. FRI was performed. Yellow circles outline sites of SPn or vehicle injections. Fluorescence images of MPO activatable biotinylated sensor (MABS) (MPO activity, red) and Sytox Green (extracellular DNA, green) as well as a merged fluorescence image (MABS plus Sytox Green) demonstrate co-localization of MPO activity with extracellular DNA, consistent with NET formation at the site of infection in wildtype mice (top row). In MPO-KO mice, extracellular DNA but not MPO activity is seen at the site of infection (bottom row). Increased levels of MPO activity and extracellular DNA are seen in the SPn injected thigh but not the saline injected thigh (*** p < 0.001, N=4 mice per group, student's t-test, experiment was replicated three times). Increased levels of extracellular DNA but not MPO activity are seen in MPO-KO mice injected with SPn or saline (*** p < 0.001, N=3 mice per group, student's t-test, experiment was replicated twice).

To assess our platform for imaging *ex vivo* tissue blocks and sections, we next induced focal cerebritis in the brains of wildtype and MPO-KO mice by intracerebral injection of salmonella (**Fig. 4A**). In the first experiment, we intravenously injected MABS and SAF-647 at 23 h after salmonella inoculation into living animals. *Ex vivo* FRI of coronal brain slices showed increased fluorescence signal consistent with MPO activity in the ipsilateral hemisphere but not the contralateral hemisphere. In contradistinction, injection of saline instead of bacteria in wildtype mice, and injection of Salmonella in MPO-knockout mice did not result in significantly elevated MABS signal.

After FRI, brain slices were snap-frozen and sections stained with anti-MPO antibody and with DAPI as a nuclear counterstain (**Fig. 4B**). Interestingly, in an area of normal appearing brain a short distance from the region of cerebritis in the ipsilateral hemisphere, we detected cells that stained positively for MPO protein but demonstrated no appreciable MPO activity. Within the region of cerebritis, we detected both MPO protein and MPO activity, but the two did not completely overlap. Sections from MPO-knockout mice showed no fluorescent signal related to either MPO protein or MPO activity.

Next, we sought to demonstrate simultaneous imaging of more than one probe by imaging neutrophil extracellular trap (NET) formation* in vivo* in a mouse model of bacterial cellulitis by injecting both wild type and MPO-KO mice with Streptococcus pneumonia (SPn), and subsequent imaging both MPO activity with MABS and extracellular DNA with Sytox Green. Sytox green is a widely utilized DNA intercalating dye with high binding affinity and has been used in detection of NETosis 46-48. We detected increased fluorescence signal of both MABS and Sytox Green in thighs injected with SPn, but not in the contralateral thigh injected with PBS only and MPO-knockout mice (**Fig. 4C**). Areas positive for MPO activity corresponded to areas of extracellular DNA (Sytox Green), suggesting the presence of NETs at the site of SPn induced bacterial cellulitis.

## Discussion

Inflammation not only damages tissues but can also bring about repair. The roles of the inflammatory cells are dynamic and can change depending on the stimuli 49-51. Such changes are challenging to assess using *in vitro* methods that may alter the characteristics of the cells and prevent longitudinal studies. While *in vivo* imaging is helpful to study inflammation without disturbing tissues, current imaging probes focus on assessing activated inflammatory cells (e.g., ^18^F-fluorodeoxyglucose, translocator protein) that can participate in both damage and repair 52. In contradistinction, MPO is expressed by neutrophils and pro-inflammatory M1-type macrophages but not by reparative M2-type macrophages 1-4, 35. MPO-derived reactive species have been demonstrated to cause damage in many inflammatory disorders 8-16. Thus, imaging MPO activity in living organisms would provide a longitudinal reporter of the damaging inflammatory response and its modulation by novel therapies.

In the present study, we describe an activatable imaging platform (MABS) for imaging MPO activity. This platform can be used to sensitively and specifically map MPO activity in different applications and scales, including both living animals as well as tissue specimens and sections. Furthermore, MABS allowed for molecular CT imaging using streptavidin-gold conjugated nanoparticles, a relatively underutilized modality in molecular imaging. In a series of proof-of-concept experiments we detected MPO activity in a variety of mouse models of infection and inflammation.

MPO catalyzes the formation of hypochlorous acid from hydrogen peroxide (H_2_O_2_), which in turn is generated during the respiratory burst by dismutation of superoxide anion. MPO can also oxidize phenols and indole derivatives through one-electron transfer mechanisms to form radicals that either oligomerize or cross-link with tyrosine-containing proteins 1. In the presence of MPO, the 5-hydroxyindole moieties are oxidized, which results in cross-linking to tyrosine residues in proteins. Free, unactivated MABS has a short blood half-life of less than 2 min. In contradistinction, in Matrigel, fibrinogen-binding, and disease model experiments, cross-linked and thus immobilized MABS remain at the sites of MPO activity. Both washout of unactivated probes and retention of the activated probes result in high specificity and high signal-to-noise ratio.

We chose SAF-647 as a secondary detection agent because of its far-red fluorescence offers better tissue penetration and photostability. This approach also adds capabilities for multimodal molecular imaging. Streptavidin can be tagged with different fluorochromes of different excitation and emission spectra to allow parallel imaging of different targets in the same subject, in our case, extracellular DNA with Sytox Green. MABS can also be combined with streptavidin-tagged gold nanoparticles for CT imaging (**Fig. 3B**), radionuclides such as ^18^F or ^64^Cu labeled streptavidin for PET imaging 53, or streptavidin-labeled iron oxide nanoparticles for MRI. The high specificity and extremely strong bond (biotin-streptavidin is the strongest known non-covalent bond) of biotin and streptavidin ensure low background signal and high specificity on imaging. We illustrated the versatility of MABS as a platform for MPO imaging in fluorescence and CT imaging at multiple scales and in both living organisms (**Figs. 2-4**), tissue specimen (**Fig. 4A**), and sections (**Fig. 4B**). MABS represents a complementary approach to other activatable MPO probes. While MPO-Gd, an activatable MRI probe 32, and ^18^F-MAPP, an activatable PET probe for MPO 54 are both potentially translatable for single modality clinical imaging, the strength of MABS is its versatility as an imaging platform.

MABS allowed investigation of both MPO activity and protein levels simultaneously. In a model of bacterial cerebritis, by comparing tissue sections incubated with MABS or an anti-MPO antibody, we made the surprising finding that while MPO activity is only elevated in the vicinity of the Salmonella injection site, normal appearing brain a little further away demonstrated strong MPO protein expression but no MPO activity. The most likely explanation for our findings is that MPO expressing cells such as neutrophils and monocytes were recruited to the brain in a state of acute infection. However, only upon further activation (e.g., by bacteria or inflammatory cytokines) was MPO released into the extracellular space 23. Hence, evaluation of MPO protein as a surrogate marker for MPO activity will likely result in not only overestimation of MPO activity, but also may implicate MPO in areas where it may be an inactive bystander instead of an active contributor. Thus, imaging both MPO activity and protein are likely necessary to tease apart the roles MPO plays in different diseases. As such, in addition to *in vivo* imaging, our imaging platform can also be an invaluable *ex vivo/in vitro* tool for histopathological investigations to improve our understanding of oxidative stress and inflammation.

NETs have only recently been described, and increasing evidence points to involvement in not only infection 19, but also thrombosis and autoimmunity 55. Hitherto, research on NETs outside of *in vitro* systems is conducted by either post-mortem histological assessment or fluorescence microscopy. We add a tool to visualize NETs at the macroscopic level by co-injecting MABS with Sytox Green to image sites of MPO activity and extracellular DNA, two defining molecular components of NETs 56. Co-localization of these two markers thus strongly suggests NET formation *in vivo.*

We found in the MPO implant experiment that no significant signal difference was observed from mice with normal chow and mice with biotin-free chow (**Fig. 2B**). Therefore, no endogenous biotin-blocking is required to conduct the experiments in living animals. However, using streptavidin does have limitations, including increased immunogenicity and accumulation in the kidneys 57, 58.

In conclusion, the MABS MPO imaging platform can specifically detect MPO activity *in vivo* and *ex vivo* at relevant biological concentrations, as validated in proof-of-concept murine disease models using both molecular fluorescence and CT imaging. Importantly, MPO activity and MPO protein may represent different pathophysiological events and should both be evaluated when investigating the effects and importance of MPO and the innate immune response. Because of its versatility, MABS can be used to uncover biology and pathology related to MPO activity from macroscopic studies *in vivo* to microscopic scales *in vitro*/*ex vivo* settings in the same animals to ensure that the process observed are not altered by tissue processing or by the use of surrogate markers that may not reflect MPO activity. Furthermore, the ability to change secondary imaging probes allows for selection of complementary imaging modalities with other imaging probes to avoid cross-talk that otherwise could result if two imaging probes are used for the same modality. Finally, imaging MPO activity would be useful to evaluate emerging therapies targeting active inflammation in many diseases in which MPO is implicated, such as cardiovascular diseases 8, 9, stroke 12, and multiple sclerosis 13, 14.

## Supplementary Material

Supplementary figures and tables.

## Abbreviations

CFA: complete Freud's adjuvant
DAPI: 4', 6-diamidino-2-phenylindole
FMT: fluorescence molecular tomography
FRI: fluorescence reflectance imaging
GNPs: gold nanoparticles
GOX: glucose oxidase
MABS: MPO-activatable biotinylated sensor
MPO: myeloperoxidase
NET: neutrophil extracellular trap
RFU: relative fluorescence units
ROIs: regions of interest
ROS: reactive oxygen species
SAF-647: streptavidin AlexaFluor-647
