# Supplementary material for: A versatile imaging platform with fluorescence and CT imaging capabilities that detects myeloperoxidase activity and inflammation at different scales
Source: Theranostics. 2019 Oct 12;9(25):7525–36. doi: 10.7150/thno.36264 (PMC6831463; doi:10.7150/thno.36264)
Supplement: Supplementary file 1 — Supplementary figures and tables. [file thnov09p7525s1.pdf]

Supplementary Material

Compound characterization by ^{1}H, ^{13}C NMR and LC-MS

Compound **2**. ^{1}H NMR (500 MHz, DMSO) δ 12.48 (s, 1H), 10.48 (s, 1H), 8.59 (s, 1H), 7.11 (d, J = 8.0 Hz, 1H), 7.02 (d, J = 2.0 Hz, 1H), 6.93 (D, J = 8.5 Hz, 1H), 6.81 (d, J = 2.0 Hz, 1H), 6.57 (dd, J1 = 8.5 Hz, J2 = 2.0 Hz, 1H), 4.09 (m, 1H), 3.00 (dd, J1 = 28 Hz, J2 = 5Hz, 1H), 2.86 (dd, J1 = 28 Hz, J2 = 5Hz, 1H); 1.32 (s, 9H); ^{13}C NMR (125 MHz, DMSO) δ 174.0, 155.4, 150.2, 130.6, 127.7, 124.1, 111.7, 111.2, 109.1, 102.0, 78.0, 54.3, 28.2, 26.9; LCMS found m/z: 321.3 (M+1).

Compound **3**. ^{1}H NMR (500 MHz, DMSO) δ 10.45 (s, 2H), 8.56 (s, 1H), 8.54 (s, 1H), 7.89 (t, 1H), 7.10 (d, J = 8.5 Hz, 1H), 7.09 (d, J = 9.0 Hz, 1H), 7.0 (m, 2H), 6.88 (d, J = 1.5 Hz, 1H), 6.83 (d, J = 2.0 Hz, 1H), 6.67 (d, J = 8.5 Hz, 1H), 6.57 (m, 2H), 4.12 (m, 1H), 3.26 (m, 2H), 2.96 (dd, J1 = 14.5 Hz, J2 = 4.5 Hz, 1H), 2.79 (dd, J1 = 14.5 Hz, J2 = 4.5 Hz, 1H), 2.66 (m, 2H), 1.32 (s, 9H); ^{13}C NMR (125 MHz, DMSO) δ 171.8, 155.1, 150.15, 150.13, 130.8, 130.6, 128.1, 127.8, 123.9, 123.0, 111.6, 111.4, 111.2, 111.1, 110.7, 109.3, 102.5, 102.2, 77.9, 55.0, 40.1, 28.2, 28.0, 25.2; LCMS found m/z: 479.4 (M+1).

Compound **1**. ^{1}H NMR (500 MHz, DMSO) δ 10.5 (s, 1H), 10.4 (s, 1H), 7.96 (t, 1H), 7.89 (d, J = 8.0 Hz, 1H), 7.10 (d, J = 8.5 Hz, 1H), 7.09 (d, J = 8.5 Hz, 1H), 6.99 (m, 2H), 6.88 (d, J = 2.0 Hz, 1H), 6.82 (d, J = 2.0 Hz, 1H), 6.57 (m, 2H), 6.39 (b, 2H), 4.47 (m, 1H), 4.26 (m, 1H), 4.04 (m, 1H), 3.26 (m, 2H), 3.00 (m, 2H), 2.78 (m, 3H), 2.65 (m, 2H), 2.07 (m, 2H), 1.55 (m, 1H), 1.42 (m, 3H), 1.20 (m, 2H); ^{13}C NMR (125 MHz, DMSO) δ 173.6, 173.2, 164.4, 151.8, 132.5, 132.3, 129.7, 129.5, 125.6, 124.8, 113.3, 113.1, 112.9, 112.8, 112.4, 111.0, 104.2, 103.9, 62.6, 60.9, 57.0, 54.9, 36.6, 29.7, 29.6, 29.5, 27.1, 26.9, 26.8, 26.0; LCMS found m/z: 605.3 (M+1).

Compound **1'**. ^{1}H NMR (500 MHz, DMSO) δ 10.79 (s, 1H), 10.77 (s, 1H), 8.03 (m, 1H), 7.94 (d, J = 8.0 Hz, 1H), 7.58 (d, J = 8.0 Hz, 1H), 7.52 (d, J = 8.0 Hz, 1H), 7.31 (m, 2H), 7.10 (m, 2H), 7.04 (m, 2H), 6.96 (m, 2H), 6.39 (s, 1H), 4.50 (m, 1H), 4.25 (m, 1H), 4.03 (m, 1H), 3.30 (m, 2H), 3.05 (m, 1H), 2.98 (m, 1H), 2.88 (m, 1H), 2.75 (M, 2H), 2.54 (d, 1H), 2.05 (m, 2H), 1.53 (m, 1H), 1.41 (m, 3H), 1.18 (m, 2H); ^{13}C NMR (125 MHz, DMSO) δ 172.9, 172.5, 163.7, 137.1, 137.0, 128.3, 128.1, 124.4, 123.6, 121.8, 121.7, 119.5, 119.2, 119.1, 119.0, 112.7, 112.3, 112.2, 111.3, 61.9, 60.1, 56.3, 54.3, 35.9, 29.0, 28.8, 28.7, 26.1, 26.0. LCMS found m/z: 573.4 (M+1).

Supplementary Figures

A

n	Annotation	Observed	Theoretical	mass accuracy (ppm)
1	$[M+H]^+$	605.2540	605.2541	-0.17
1	$[M-H]^-$	603.2398	603.2395	0.50
2	$[M+H]^+$	1207.4840	1207.4852	-0.99
2	$[M-H]^-$	1205.4698	1205.4707	-0.75
3	$[M-H]^-$	1807.6966	1807.7018	-2.88
3	$[M-2H]^{2-}$	903.3456	903.3473	-1.88

B

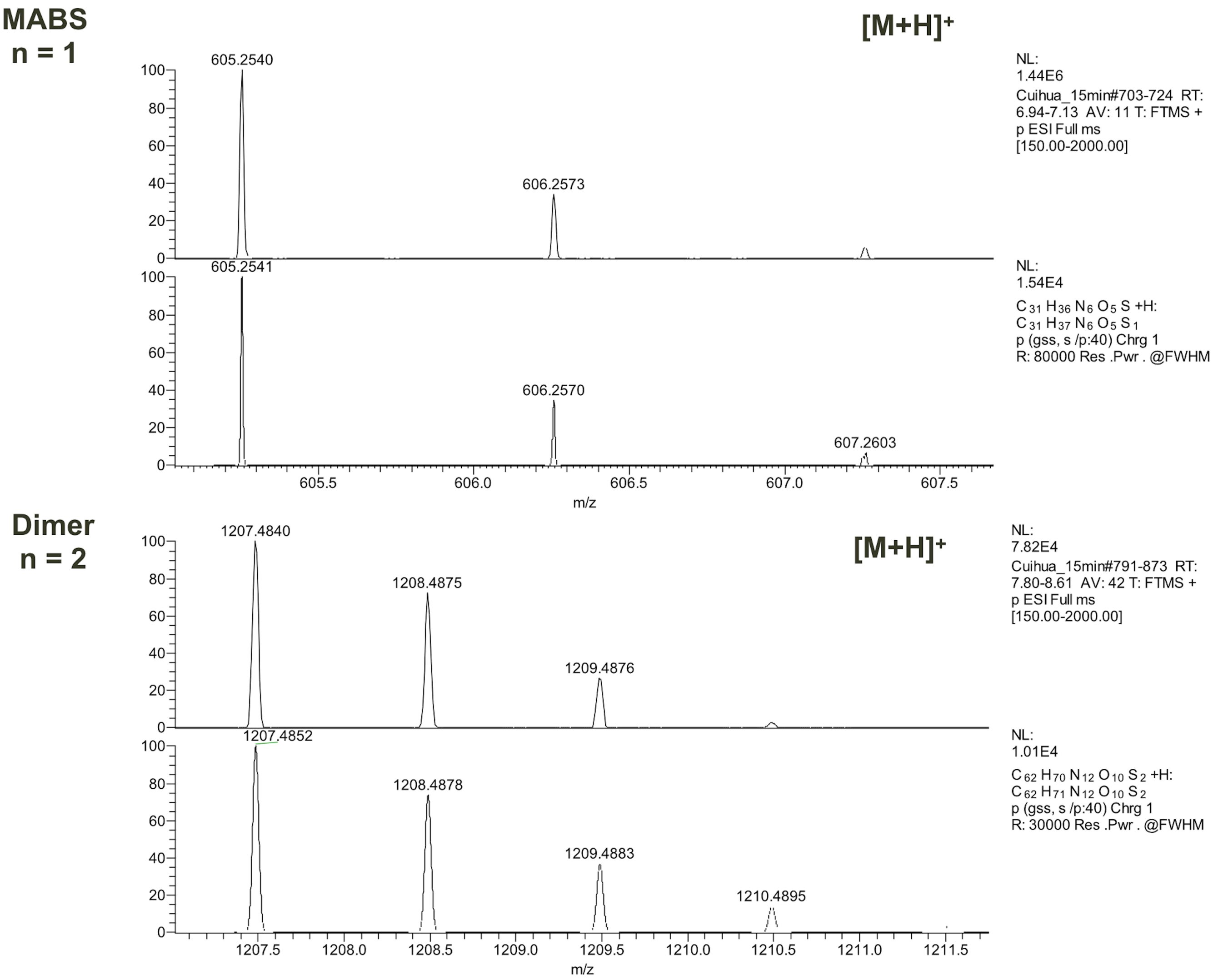

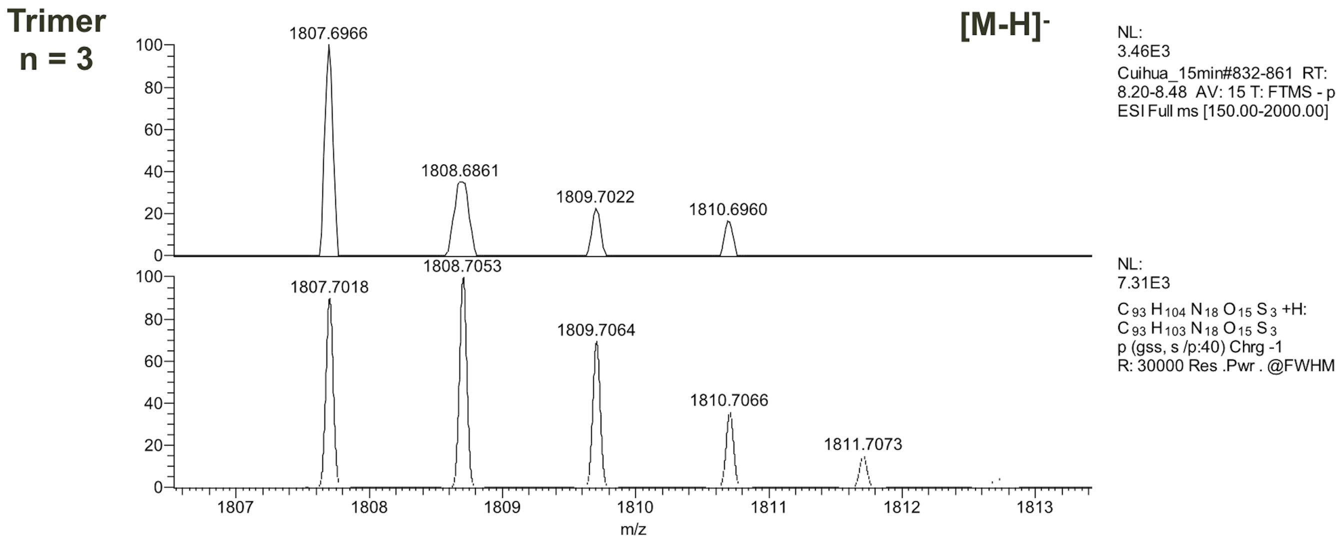

Figure S1. Identification of oligomer formation of MABS after activation by MPO with high-resolution LC-MS. (A) Result summary of MABS and its oligomers from both positive ion mode and negative ion mode detection (n=1, MABS; n=2, dimer; n=3, trimer). (B) Representative chromatograph of high-resolution LC-MS of MABS, its dimer and trimer (upper: experimental m/z; lower: theoretical m/z).

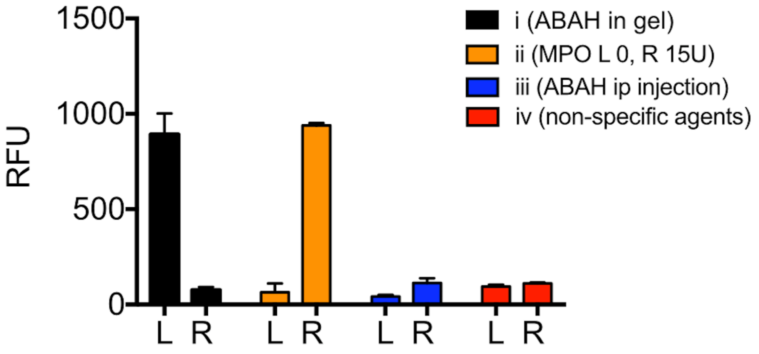

Figure S2. Quantification of Fig. 2C for validation of specificity of MABS in Matrigel implant experiment. i. ABAH embedded on right side; ii. 0U and 15U of MPO on left and right side, respectively; iii. ABAH i.p. injected; iv. Non-specific agents administered. (N=3)

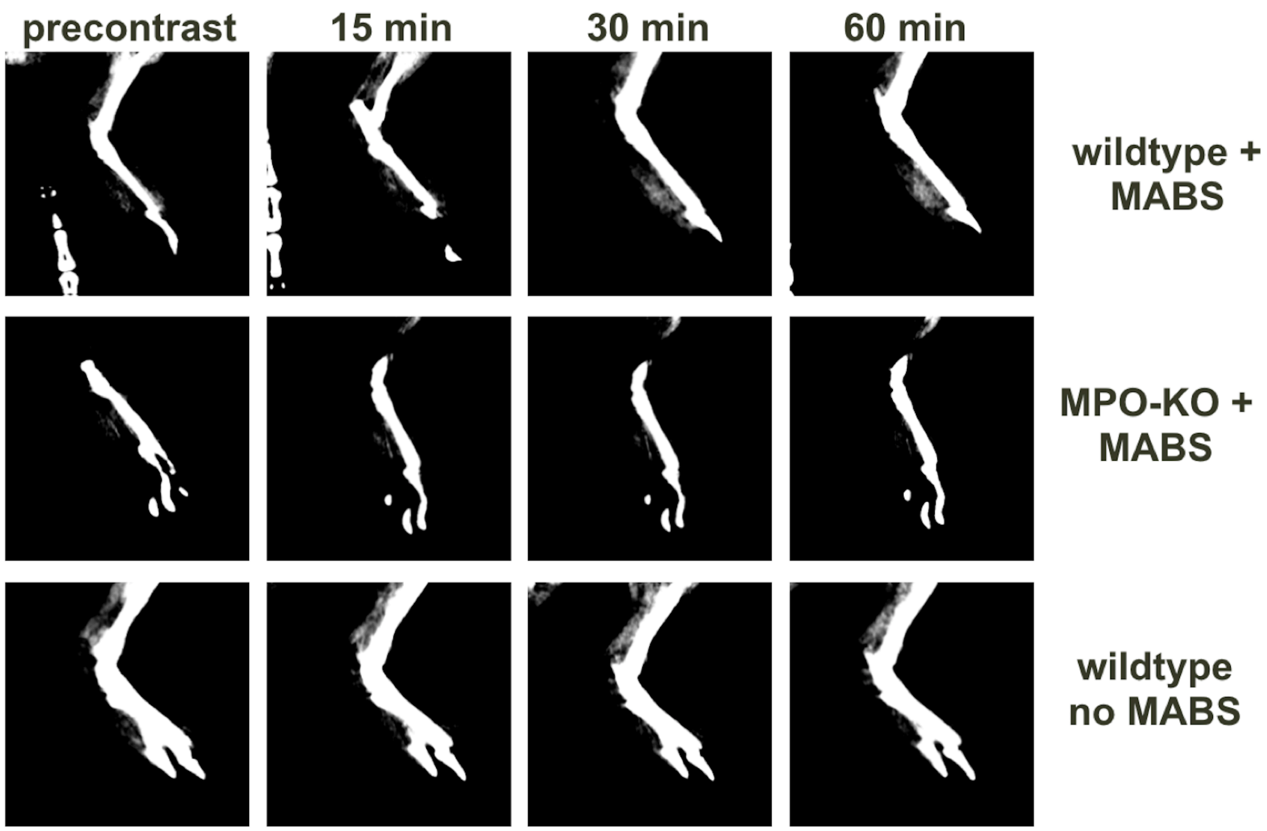

Figure S3: Representative gray-scale CT images of paw inflammation induced by complete Freund's adjuvant.
